# SAFARIS: a spatial analytic framework for pest forecast systems

**DOI:** 10.3389/finsc.2023.1198355

**Published:** 2023-07-06

**Authors:** Yu Takeuchi, Amber Tripodi, Kellyn Montgomery

**Affiliations:** ^1^ Center for Integrated Pest Management, North Carolina State University, Raleigh, NC, United States; ^2^ Plant Pest Risk Analysis, Science & Technology, Plant Protection and Quarantine, Animal and Plant Health Inspection Service, United States Department of Agriculture, Raleigh, NC, United States; ^3^ Phytosanitary Advanced Analytics Team, Business and Employee Services, Plant Protection and Quarantine, Animal and Plant Health Inspection Service, United States Department of Agriculture, Raleigh, NC, United States

**Keywords:** non-native pests, predictive modeling, spatial analytic framework, phytosanitary management, oak ambrosia beetle, spongy moth

## Abstract

Non-native pests and diseases pose a risk of economic and environmental damage to managed and natural U.S. forests and agriculture. The U.S. Department of Agriculture (USDA) Animal and Plant Health Inspection Service (APHIS) Plant Protection and Quarantine (PPQ) protects the health of U.S. agriculture and natural resources against invasive pests and diseases through efforts to prevent the entry, establishment, and spread of non-native pests and diseases. Because each pest or disease has its own idiosyncratic characteristics, analyzing risk is highly complex. To help PPQ better respond to pest and disease threats, we developed the Spatial Analytic Framework for Advanced Risk Information Systems (SAFARIS), an integrated system designed to provide a seamless environment for producing predictive models. SAFARIS integrates pest biology information, climate and non-climate data drivers, and predictive models to provide users with readily accessible and easily customizable tools to analyze pest and disease risks. The phenology prediction models, spread forecasting models, and other climate-based analytical tools in SAFARIS help users understand which areas are suitable for establishment, when surveys would be most fruitful, and aid in other analyses that inform decision-making, operational efforts, and rapid response. Here we introduce the components of SAFARIS and provide two use cases demonstrating how pest-specific models developed with SAFARIS tools support PPQ in its mission. Although SAFARIS is designed to address the needs of PPQ, the flexible, web-based framework is publicly available, allowing any user to leverage the available data and tools to model pest and disease risks.

## Introduction

Climate and weather-driven forecast systems that incorporate biological drivers are important tools for a wide range of applications. Pest and production forecast systems are widely used by farmers not only to predict suitable timing for planting, spraying pesticides, and harvest schedules, but also to advance more cost-effective production approaches. Regulatory agencies also use pest forecast systems to establish early warning systems before non-native pests arrive, help eradicate new arrivals, or manage non-native species that are already established invaders.

In the United States, the U.S. Department of Agriculture, Animal and Plant Health Inspection Service, Plant Protection and Quarantine (PPQ) leads the Federal-level response to invasive species that affect plant health. PPQ aims to protect American agriculture and natural resources against the entry, establishment, and spread of economically and environmentally significant plant pests and diseases (1). Within PPQ, the Plant Pest Risk Analysis group in Science and Technology provides risk assessments, scientific analyses, and other information to support PPQ regulatory decisions and activities, many of which include spatial analyses that evaluate the likelihood and consequences of pest and disease establishment. For example, they provide spatial analyses that inform the Cooperative Agricultural Pest Survey (CAPS) community of when and where to survey for targeted high priority pest species (2). CAPS is a network of state and federal cooperators that focuses on early detection to prevent new non-native plant pests from becoming established. Spatial analyses help other PPQ programs quickly address new detections, manage pest spread, determine trade risks, and other phytosanitary issues.

A number of systems and techniques already exist to predict suitable areas for plant pest and disease establishment based on various factors, including climatic variables (3, 4). Nevertheless, researching, selecting, processing, and standardizing climatic variables from various sources to construct models is time consuming. In addition, predicting invasive pest behavior in natural (and often novel) environments is complex and involves uncertainty; therefore, it is often difficult to determine which approaches might give the most realistic and practical results. Ensemble modeling approaches are commonly utilized in this context, especially for newly discovered pests whose biology is not well known (5–7). When a species is in the early stages of invasion, the relationship between the species and the environment is particularly difficult to understand, and capturing uncertainty can help decision-makers understand what the potential risks may be (8, 9).

In this paper, we describe the Spatial Analytic Framework for Advanced Risk Information Systems (SAFARIS), a pest risk forecasting framework developed for regulatory agencies. We developed this framework to establish a consistent, tractable, and comprehensive environment that supports pest models driven by abiotic and biotic factors. These models output pest forecasts for local, regional, national, or global phytosanitary pest and disease management. The framework also integrates tools that specifically address uncertainty to interpret and communicate it for policy and area-wide management. Establishing a common framework to run a variety of models has numerous advantages, such as enabling valid comparisons between different models and improving the role and value of models for informing policy. We built SAFARIS to support organizations responsible for sanitary and phytosanitary management such as PPQ, although the system can be adapted by any organization that wishes to use models driven by climatic, weather, and biological factors. In this paper, we describe how we built SAFARIS and demonstrate how it is used to create climate suitability maps and pest forecasts to support PPQ programs.

## Materials and methods 

### Components of SAFARIS

**Figure 1:**
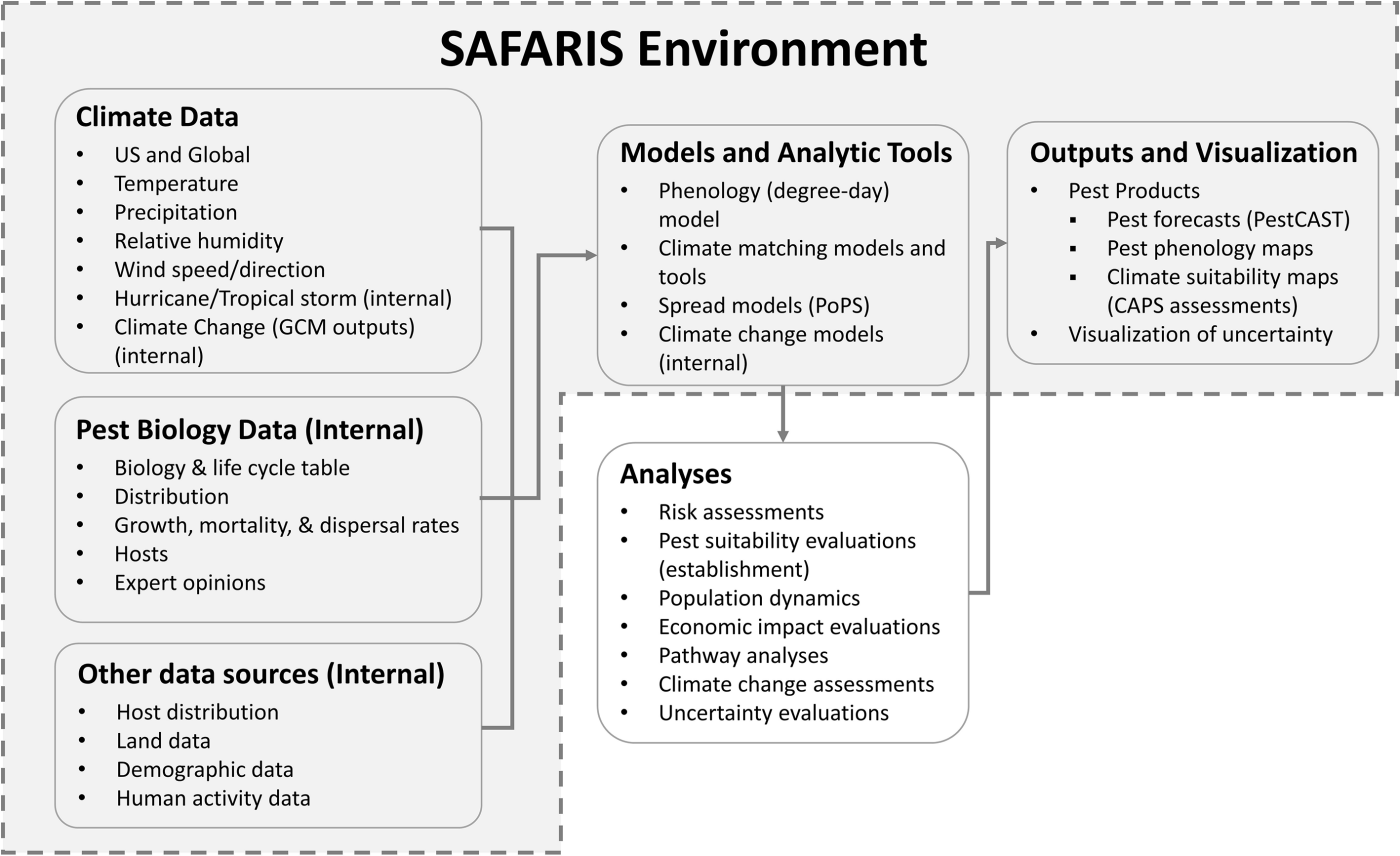
SAFARIS framework environment. Commonly used input data drivers for models and analytic tools available within SAFARIS are climate data, pest biology data, and human activity data. These datasets are connected to models and analytic tools such as phenology model, climate matching models and tools, spread models, and climate change models. The spread model called Pest or Pathogen Spread Model (PoPS) runs externally using data drivers within SAFARIS. These models and analytic tools are regularly used to analyze PPQ concerned pests and diseases. Some assessments are visualized using interactive mapping systems within SAFARIS as Pest Products.

SAFARIS is a web-based spatial analytic framework and uses a multi-compartment design focused on supporting multiple types of models or applications, including phenology models, climate matching models and tools, spread models, and climate-change models. In addition to integrating relevant and adaptable pest models, SAFARIS stores associated data drivers required to run the models using standardized formats that feed directly into the model tools. Thus, SAFARIS is composed of standardized, pre-processed climate data, internal pest biology data and other non-climate data sources, predictive models and tools, and model output visualizations (**Figure 1**). Together, these components allow custom analyses on-demand with outputs that are archived in SAFARIS and easily shared. SAFARIS houses historical weather data that can be used to analyze long-term trends and create forecasts based on historical weather averages. Alternatively, real-time weather data can be used to drive models to create more accurate within-year forecasts that are based on current weather patterns. The framework is scalable for hosting an unlimited number of models, but it is most advantageous for models which can leverage the data pipeline provided by SAFARIS by using the drivers already available and consistently formatted in the framework. SAFARIS is open to the public and accessible via a web-browser at https://safaris.cipm.info. Users have access to most tools, models, and climate data; however, some are internal and only available to registered users.

We developed SAFARIS to allow users to choose among different climate databases and geographical target areas (e.g., specific area within a country, specific country, or global). The climate databases provide 1) historical climate data, 2) short-term weather forecasts (e.g., 7-day or 30-day forecasts) and 3) long-term climate projections (e.g., downscaled General Circulation Model (GCM) outputs) (**Supplemental Table 1**). SAFARIS selects the climate data set appropriate for the temporal and spatial resolution of the selected model. For example, when a user selects the Temperature Mapping Analytic Tool to determine suitable areas for a pest’s winter survival, the model is automatically connected to monthly climate datasets. However, because biological development may happen in less than one month, the Phenology Model creates forecasts with daily minimum and maximum temperatures to calculate developmental rates for a particular pest based on user-specified thresholds. The framework supports user-selected time-step specifications to address the associated temporal variability in models. In addition, a user can run models using historical climate data, short-term weather forecasts, and downscaled GCM climate data to create pest predictions that can vary from near real-time forecasts (e.g., two-week outlooks) to short-term (several months) and long-term (years to decades) forecasts.

### Models and analytic tools

The SAFARIS framework currently includes phenology and spread forecasting models and many climate-based analytical tools commonly used in phytosanitary applications. Each model or tool has a straightforward template linked to necessary climate data drivers that enables a user to select custom parameters. We list the currently available web-based models and tools within SAFARIS that are commonly used in pest risk assessments (**Supplemental Table 2**). SAFARIS is frequently updated with new tools as they are developed in-house or through collaborations with other researchers (10, 11).

SAFARIS provides phenology models that can be used to predict the timing of an organism’s developmental stages. For example, environmental temperatures directly affect the rate of organism growth and development if other resources are not limited (12). Phenology “degree day” models calculate a standardized estimate of heat exposure over time (heat units or degree days) for the organism of interest. Many species require a certain amount of heat exposure to develop from one stage to another. This required heat over time is expressed in physiological time because each organism requires a different amount of heat for development, and because environmental temperatures vary each day (12, 13). Physiological time is calculated by multiplying cumulative developmental time by the temperature above a declared developmental threshold and is therefore expressed as degree-days (12, 13).

SAFARIS also provides climate analytic tools to determine the occurrence of specific climate conditions that support pest growth based on the historical and forecasted weather data. Outputs from these tools are used in environmental suitability models, that determine suitable geographic areas for pest establishment by evaluating climatically suitable conditions for a given pest’s growth and often add **Supplemental Information** such as host species distribution, host species availability, elevations, and land cover types (4, 14–18). Overall, an invasion ecology model underlies this approach, which focuses on climate (environment) suitability, host availability, and pest presence (19).

### Outputs and visualization

Phenology model and analytic tool results in SAFARIS are distributed as maps in netCDF format based on user-specified criteria. Processing times for most results will be between a few minutes and a few hours. The resolution and extent of the data, the analysis process and network bandwidth may affect the timeliness of results. To account for possible delays in server exchanges, users are notified by email when the framework compiles requested maps. In addition to having access to raw data drivers, users can view the input data information and output maps on the website and can download the output maps as rasters in netCDF format that users can import into their own GIS systems.

In terms of specific outputs, the Phenology Model generates a raster of degree-days accumulated during a user-specified time frame based on user-specified developmental threshold temperatures. These models depict potentially suitable areas where enough physiological time has passed for a given species to complete a single generation or specified developmental stages.

The Analytic Tools for evaluating weather data generate two types of outputs: 1) a raster in which each cell value represents the number of days that meet user specified conditions (i.e., pest growth requirement(s) for specific consecutive days) during the study period and 2) a raster of binary values indicating whether the user specified conditions have been met during the study period. The weather conditions examined can include temperature (daily maximum, daily minimum, and daily average), and relative humidity (daily minimum, daily maximum, and daily average). The Precipitation Mapping Analytic Tool generates 1) accumulated precipitation during the user specified period and 2) a raster of binary values indicating whether the user specified conditions have met during the specified period. Additionally, the Global Plant Hardiness Zones tool identifies where organisms could be established by matching zones with organisms’ distribution areas. These analytic tools are useful to understand if areas may support plant pest growth and to determine the likelihood of pest activity and establishment.

Outputs from Models and Analytic Tools can be further analyzed outside of SAFARIS. For example, the outputs from the Temperature Mapping Analytic Tool and the Precipitation Mapping Analytic Tool can be combined to identify areas where both temperature and precipitation requirements meet to determine pest suitable areas using software such as R and ArcGIS Pro.

Since the models and input data drivers are embedded in SAFARIS, pest forecasts can be automated and visualized within its framework. SAFARIS produces and houses many Pest Products, which are the outputs of other pest-specific models relevant to current PPQ phytosanitary concerns (**Figure 1**). At present, this includes map products for 41 species. The types of maps generated for the pests vary with species and data availability; however, all maps are intended to help users understand where pests have the potential to establish, when to expect pest activity, and when and where pests could have an impact within the United States. Three types of Pest Products are currently available: PestCAST and CAPS suitability maps, which are dynamic, interactive maps showing the most up-to date information available, and Field Operations Weekly Maps, which are static PDF files. These Pest Products directly support PPQ and state field operation activities and are easily shared to a wide audience.

## Application

### Use case: climate suitability maps for pest surveillance

The Cooperative Agricultural Pest Survey (CAPS) program conducts non-native plant pest surveys through a national network of cooperators and stakeholders to protect American agriculture and natural resources (2). Since CAPS pests do not occur in the United States, we generate climate suitability maps for CAPS pests using SAFARIS that help support survey planning and prioritization by showing where pests could establish in the United States. Because CAPS surveys are tasked with early detection, these models are designed to incorporate all areas that could potentially support pest establishment, including marginally suitable areas, and only exclude areas that we are certain would be unsuitable. The CAPS suitability models are mechanistic and customized with information specific to the biology of a pest, such as known temperature tolerances, moisture requirements, or physiological requirements for growth. Once a custom suitability model is developed for a pest, SAFARIS uses historical weather data to predict where the pest could establish and displays the result as an interactive map on the website (20, 21). Static maps showing model results, along with a methodology document that describes model parameters and assumptions, are available for download on the website as PDFs, and technical users can download the netCDF maps to use in their own spatial analyses. These maps help CAPS coordinators prioritize which pests to survey for in their area and coordinate survey timing.

#### Example: CAPS pest suitability assessment for oak ambrosia beetle

Oak ambrosia beetle (OAB), *Platypus quercivorus* (Murayama, 1925), is found in temperate, subtropical, and tropical forests in India, Indonesia, Papua New Guinea, Taiwan, and Japan. Its hosts include many species in the Fagaceae family, but it may attack some species within Aquifoliaceae, Lauraceae, Rosaceae, Cupressaceae and Taxodiaceae (22, 23). It develops in the inner bark and wood of host trees and feeds on a symbiotic fungus, *Raffaelea quericivora*. This fungus is the causal agent of Japanese oak wilt, which causes severe damage in susceptible oak species in Japan. If North American oaks are susceptible to Japanese oak wilt, establishment of the OAB in the United States has potential to cause significant mortality and economic and environmental damage. Davis, French and Venette (22) identified twenty-eight potential host species of *Quercus* for OAB in the continental United States.

We reviewed scientific literature to identify climate conditions required for pest development. The lower lethal temperature was identified as the lowest supercooling point (-20.3°C) measured by a lab experiment (24) minus a thermal insulation constant (1.2°C) to account for the difference between interior tree temperatures and ambient temperatures (25). This adjustment was necessary because ambient temperature records were used in the assessment. Therefore, we modeled suitable areas using the Temperature Mapping Analytic Tool to determine the areas where daily temperatures never reached the lower lethal threshold of -21.5°C. To parameterize the phenology model, we used the work by Barker and Coop (26) from OAB monitoring data (27) and trapping data of similar bark beetle species (28). The phenology model parameters used were a lower developmental threshold temperature of 11.1°C, an upper developmental threshold temperature of 38°C, and an accumulation of 1,486 degree-days for one generation.

For each year between 2001 and 2020, areas considered suitable for pest establishment met the climate suitability criterion of daily ambient temperatures above the lower lethal threshold identified from scientific literature. We did not include the OAB phenology requirements to complete one generation as an annual requirement. OAB can have one or two generations throughout the year (27); however, some beetles may take two years to complete a life cycle (27). Therefore, we only used the lower lethal temperature as a suitability requirement and designated the areas where daily ambient temperatures were never fatal as areas suitable for establishment. We then used the Phenology Model to estimate the number of generations an area could support.

**Figure 2:**
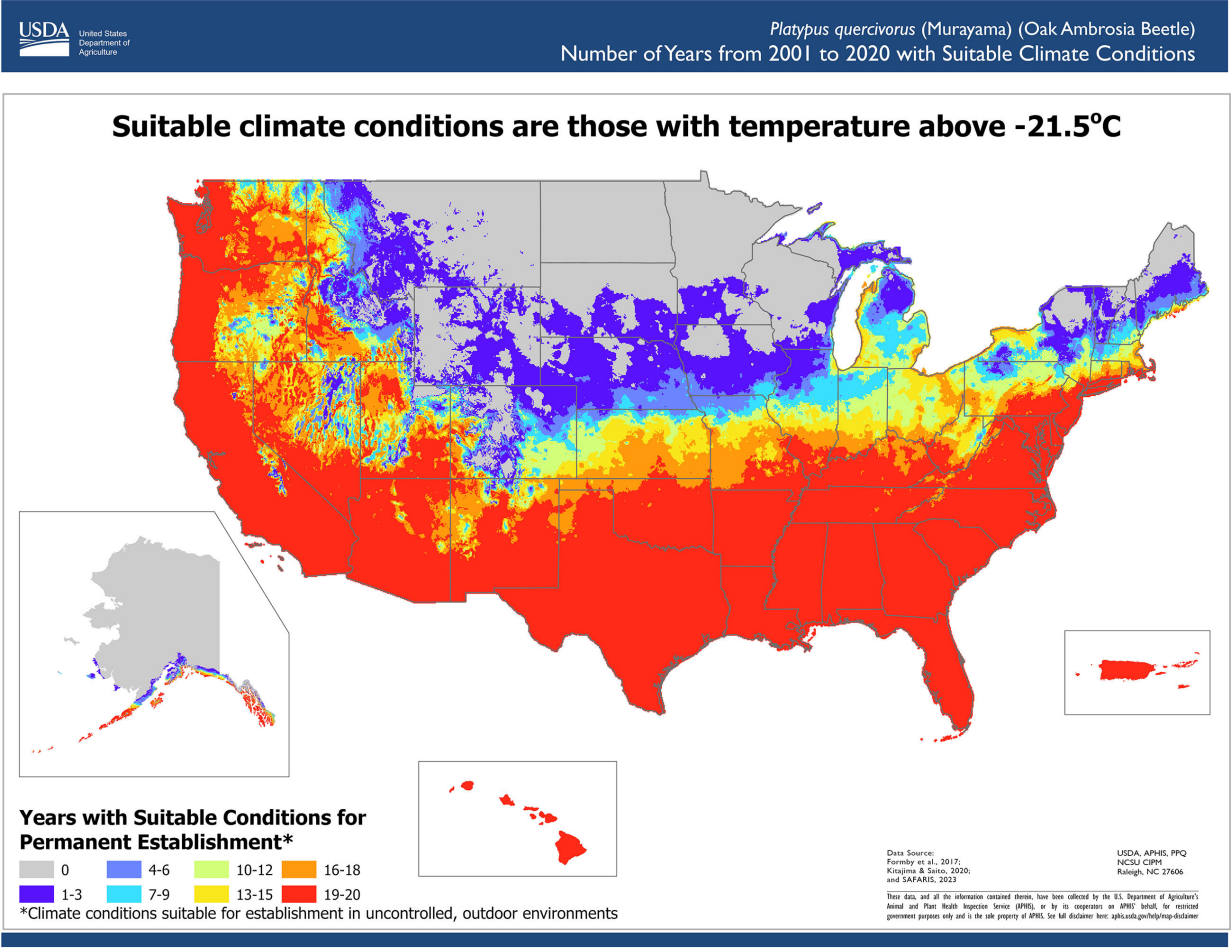
Number of years with suitable conditions for oak ambrosia beetle in the contiguous United States, Hawaii, Puerto Rico, and Alaska. Suitable condition requirements were the daily ambient temperatures were above (-21.5°C), the lower lethal temperature threshold (-20.3°C) with a tree-interior insulation factor of 1.2°C.

**Figure 3:**
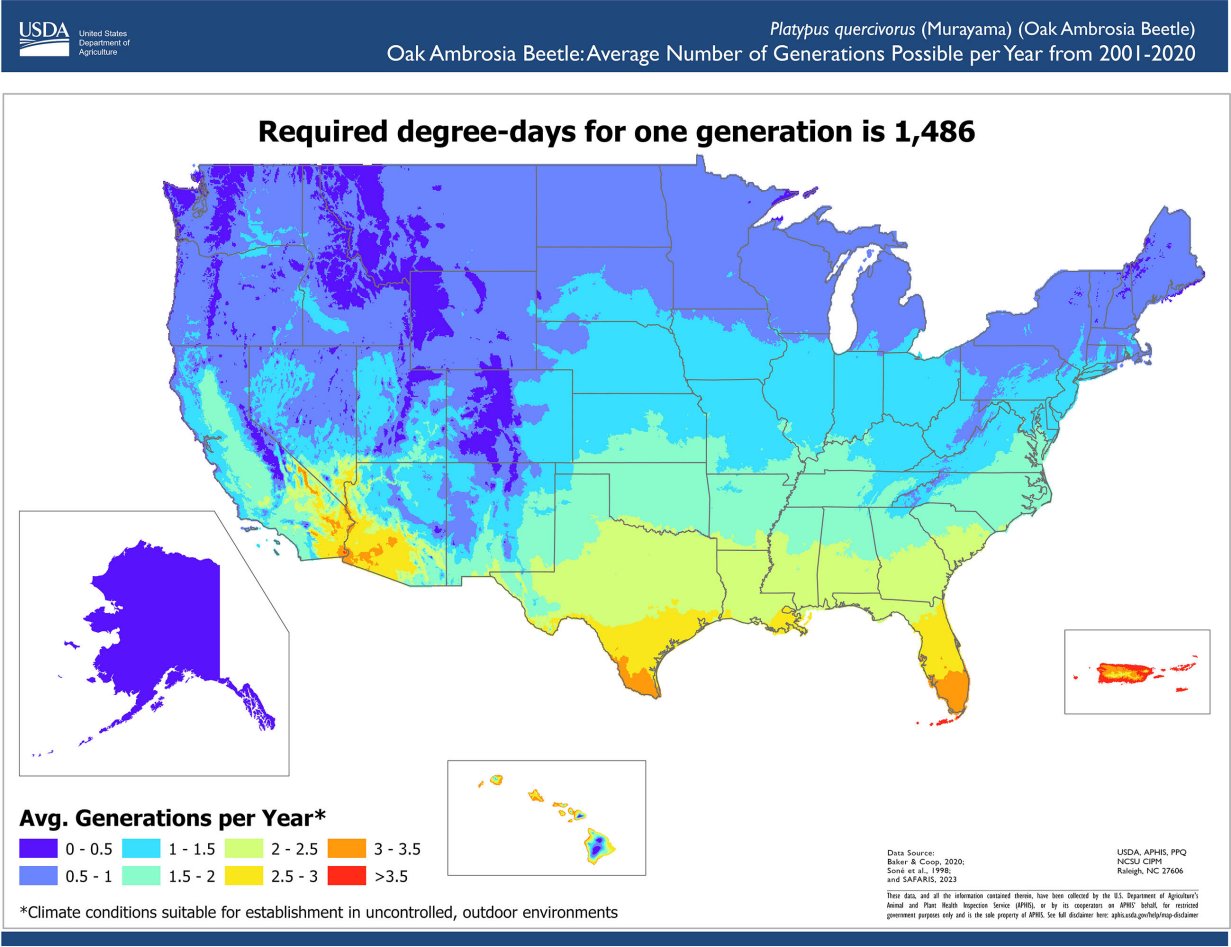
Average number of generations possible per year for oak ambrosia beetle in the contiguous United States, Hawaii, Puerto Rico, and Alaska. The development from egg to egg-laying adult requires the accumulation of 1,486 degree days.

We determined the number of years with suitable conditions for OAB in the contiguous United States, Alaska, Hawaii and Puerto Rico using twenty years of PRISM and Daymet weather data (2001–2020), resulting in a resolution of 4 km in the contiguous United States and 1km in Alaska, Hawaii, and Puerto Rico (20, 21) (**Figure 2**). The results indicated that the Southeast, Southwest, the West Coast, Hawaii, and Puerto Rico have suitable climate conditions for OAB establishment. The southern portions of Midwest and Northeast and parts of the West have suitable conditions for some years, so its ability to establish in those areas is less certain. Unsuitable areas included the northern portions of the Northeast, Midwest, and West regions. Much of the West and Northeast regions do not experience enough annual growing degree days to allow OAB to complete one generation a year; however, in those areas, OAB may overwinter and complete its life cycle the following year (**Figure 3**). Southern regions can support multiple generations per year and should be considered optimal for OAB. Since voltinism for OAB is unknown, our results indicate the potential number of generations each year. The second generation may or may not be successful in rearing its own brood, and although the evidence suggests that some beetles can take more than one year to complete their development, this has not been conclusively shown (27).

The OAB climate suitability maps are displayed using interactive web mapping interface within SAFARIS (https://safaris.cipm.info/safarispestmodel/StartupServlet?caps&pid=OAB). The interface allows users to click, pan, and zoom for further information. Location specific information can be obtained by clicking an area on the map, and users can toggle between different layers to easily visualize multiple assessment results. The web-based visualization system organizes assessment results and modeling methods in a format that is easy to share with others, either interactively on the website or in PDF format. The results of these maps help survey planners decide if their area is suitable for OAB, and if so, where they should focus their survey efforts. Additionally, GIS users can download raster files of model results to use in their own analyses. For example, survey planners may combine the map of suitable areas with a map of suitable host distribution to select the most appropriate areas to survey for a pest.

### Use case: pest forecasts for plant pests and diseases programs

PPQ regulates non-native plant pests and diseases and safeguards agriculture and natural resources from the risk associated with entry, establishment, or spread of non-native species. PPQ responds to newly detected plant pests with domestic programs designed to eradicate, suppress, or contain pests to minimize impacts (1). To support domestic programs, we have created a forecast system called PestCAST that indicates current pest stages and predicts expected pest stages for the next 7 and 30 days using near real-time weather data (PRISM Climate Group (20)), National Oceanic and Atmospheric Administration (NOAA) 7-day forecast weather data (National Digital Forecast Database), and 20-year historical data. The forecasts are updated daily using weather data obtained by SAFARIS and visualized as interactive maps to examine conditions for specific locations and compare pest status with historical records. We have used PestCAST to develop near-real time phenology predictions for seven non-native insects that are presently in the United States by collecting and summarizing published data and field observations from all over the world. These phenology models were validated and modified with U.S. field observation data and can accommodate new observation data as they become available. Each year, we calculate the accuracy of the predictions with all available observation data by determining whether the accumulated degree days on the pest detection day at the locations are within the model predictions. If not, we then further investigate the observation data and predictions to adjust degree-day requirements in the model. Therefore, as we incorporate multi-year observation data, the predictions become more accurate. For example, we use observation data to evaluate the predictive accuracy of the model for spotted lanternfly (*Lycorma delicatula*), which is now capable of predicting the timings of nymph and adult emergence within a few days from the field observation dates.

PestCAST allows users to compare the current conditions with conditions in previous years; therefore, users can understand if the current conditions (this year’s conditions) occur earlier or later than in past years. For example, users can change the forecast date by changing the Map Date to view the forecasts from previous days. The interactive mapping interface allows users to zoom in and out or click on a forecasting area to obtain detailed information on a specific location (e.g., state, county, latitude, longitude, current condition, and forecasts). The Compare feature generates accumulated degree days for selected years at the selected location to compare the current year’s accumulation with conditions in previous years and the 10-year average. This feature helps managers to make rapid decisions and target the timing of management actions more efficiently. The PestCAST predictions are used to schedule for scouting specimens, treatment applications, and public awareness when surveyors would expect to see pests. It has been used to arrange for personnel, supplies, and budgets to be in place earlier. Thus, PestCAST provides early warning information for managers and decision makers to plan for appropriate actions accordingly (G. Parra, 2023, personal communication).

#### Example: development of PestCAST for spongy moth

Spongy moth, *Lymantria dispar* (Linnaeus), is considered damaging and is regulated by many countries as a quarantine pest of concern. It was first introduced into the northeastern United States from Europe in 1869 (29). The spongy moth has become established and spread to 20 states and the District of Columbia in the eastern United States (30). The spongy moth has a wide host range, attacking over 500 species such as *Quercus*, *Carpinus*, *Alnus*, *Prunus*, *Populus*, *Gleditsia*, *Tilia*, *Corylus*, and *Robinia* (31). It is considered a major forest pest in the United States and Canada due to its ability to cause economic and environmental damage (32).

To forecast the timing of spongy moth (*Lymantria dispar* L.) adult emergence, we parameterized the Phenology Model based on the work of Sheehan (33). The parameters were: start date = January 1; accumulated degree days for the first adult emergence = 1,143; accumulated degree days for flight period = 1143 to 1528, as determined using developmental thresholds for egg, larvae, and pupae (**Table 1**). SAFARIS runs the spongy moth adult emergence phenology model daily to predict which spongy moth stages would be present on a given day, and which stages would be present over the next 7 and 30 days using near real-time weather data, 7-day weather forecasts, and historical weather data for the contiguous United States. SAFARIS automatically collects 4-km PRISM near real-time weather data and 7-day weather forecasts from the National Digital Forecast Database (20, 34) and averages daily PRISM data for the most recent 20 years to extend the forecast for another 23 days. To provide early warning guidance for spongy moth operational decisions, we are using PestCAST to produce 30-day forecasts for the beginning of adult emergence and for the period after adult emergence has ended and adults are no longer active (post–adult emergence). PestCAST generates maps daily in PDF format for easy sharing, which are available on the SAFARIS website.

**Table 1:** Growing degree day requirements for each spongy moth stage (33).

Phenology Parameter	Egg	Larvae	Pupae
Lower development threshol	3°C	7.2°C	6.6°C
Upper development threshol	38°C	41°C	41°C
Degree-days (DD)	282	583	277

**Figure 4:**
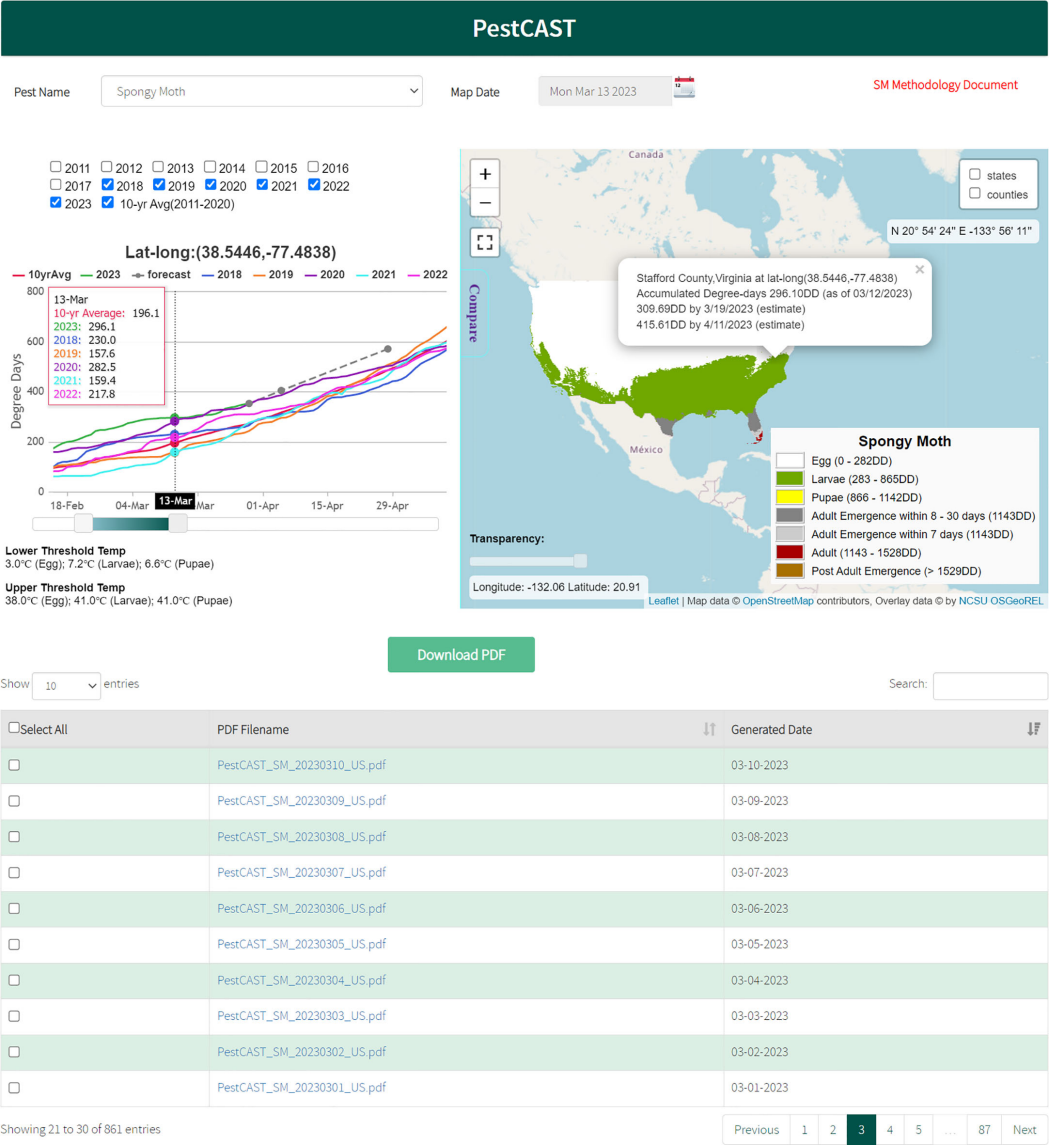
Spongy moth PestCAST: PestCAST predicts the current and the next 7- and 30-day spongy moth stages using the Phenology Model with near real-time weather information, 7-day weather forecasts, and historical weather data. The interactive mapping system allows users to obtain detailed information on specific locations and compare the current conditions with previous years by clicking the location on the map and selecting the Compare tool.

In addition, the spongy moth PestCAST forecasts are displayed using an interactive web mapping interface within SAFARIS (https://safaris.cipm.info/safarispestmodel/StartupServlet?pestcast&pid=SM). **Figure 4** displays detailed information at a location in Stafford County, Virginia as of March 13, 2023. At this location, spongy moth is currently estimated to be the larval stage, and adult emergence is unlikely to happen within the next 30 days. The graph indicates that the current (2023) year’s degree-day accumulation is faster than the last five years and the 10-year average. This means that conditions in the current year are allowing spongy moth development to occur earlier at this location, and adult emergence might happen earlier than the previous time periods.

## Discussion and future development

We have developed the SAFARIS framework to support pest and disease forecasts that are useful for management and policy-level decision making. The framework enables ‘plug and play’ functionality for weather-driven models and includes generic models that support key applications in large-scale pest and disease management. It enables the user to run models with tools that link to different weather and climate data sources easily, helping the user to understand and minimize uncertainties associated with primary input drivers. In addition, SAFARIS evaluates how climate can affect pest phenology and survival on a particular date, month, and year to understand the annual variability and trends in pest biology. Further details on the models, tools, and data drivers, along with step-by-step instructions on how to parametrize the models and tools, are available in the SAFARIS User Guide (https://safaris.cipm.info/safarispestmodel/staticfiles/safarisuserguide.pdf).

SAFARIS provides generic phenology and climate matching models that can easily be customized for a specific pest via web-based templates. It provides multiple ways to calculate degree-days (e.g., single sine method with horizontal cut-off, triangular method with vertical cut-off) that users can customize with different temperature threshold values at each pest stage. All models and tools within SAFARIS link to multiple weather/climate data sources to predict pest phenology and suitability based on historical weather conditions, near real-time weather conditions (current year), short-term future conditions, and long-term future conditions. SAFARIS allows users to combine outputs from multiple models and tools to generate applied predictions and visualize uncertainty.

Other models in SAFARIS are designed to address specific regulatory needs. The CAPS pests are not known to occur in the United States, and survey efforts are focused on early detection. Therefore, rather than reporting the likelihood of establishment, our goal is to identify all areas that could potentially support permanent establishment of CAPS pests. By designing our models to exclude areas as unsuitable only when we are certain and showing the variability among years, survey planners have the information they need to prioritize their surveys. Because we use mechanistic model approaches, CAPS pest models estimate climatic suitability sufficiently well to provide useful scientific information for regulatory decision making (35). The CAPS pest suitability use case presented here identified the current climate suitability in the United States for OAB, indicating areas suitable for OAB establishment. To incorporate the recent weather patterns in CAPS assessments, SAFARIS uses the most recent weather data for analyses because SAFARIS is linked to multiple external servers, such as NOAA’s data server and PRISM server, and automatically updates weather and climate data within SAFARIS as new data become available. This keeps our short-term predictions up to date. The PestCAST products help managers and decision-makers respond to current needs for domestic program pests in a timely fashion based on historical, current, and short-term forecast data. These SAFARIS map products have been developed to help PPQ prioritize survey efforts, respond to incursions, and allocate resources efficiently. However, SAFARIS contains global scale information and models are validated with observation data and literature on where particular pests occur in or outside of the United States. The SAFARIS framework is flexible, allowing users to construct new models quickly to address new needs as they arise.

Climate change can increase the frequency and intensity of extreme weather events. These extreme weather conditions are expected to significantly influence agricultural pests (36, 37). We are currently developing new analytic approaches using long-term projection climate models to understand the potential changes in pest establishment and impacts under future climate change. Extreme weather events affect plant pest species differently. Some pests may not have any noticeable impacts, while others might have significant impacts. Projections of future weather conditions are inherently uncertain. Therefore, SAFARIS has incorporated twenty GCMs and two Relative Concentration Pathways (RCP), which allows researchers to examine differences among predictions to characterize and communicate that uncertainty. We continue to evaluate critical information from the climate change research community to communicate with decision-makers on how these predictions should be used in decision-making processes.

The mission of the SAFARIS framework is to support decision-making processes by providing critical scientific information in a timely manner. Since PPQ often deals with pests that are not well-studied and have a lot of unknowns (i.e., lack of knowledge of how a non-native species would react to a new environment, or uncertainty about how non-native species interact with native species), understanding and quantifying uncertainty becomes important for decision-making processes. For example, evaluating the uncertainty associated with the CAPS products provided in SAFARIS is essential for increasing the validity and the accuracy of the predictions (38–40). The flexible design of SAFARIS allows users to quickly create alternate models to test model assumptions and see how adjustments to model parameters can affect the outcome.

Well-documented pest forecasts are useful; however, incorporating uncertainty information will lead to better pest management. Yemshanov, Koch and Ducey (41) demonstrated two approaches (mean-variance frontier concept and second-degree stochastic dominance rule) to evaluate uncertainties that could be incorporated into pest forecast results. These approaches could provide decision-makers with additional guidance for prioritizing pest control options (41). Because the tools provided by SAFARIS use very high data granularity, the user is able to assess variability and uncertainty associated with input data and model outputs. We are further investigating methods to combine multiple sources of uncertainty (e.g., natural variation, climate change uncertainty, and model errors) that can affect pest predictions and developing ways to effectively communicate the uncertainty associated with spatial assessments to decision-makers.

SAFARIS will continue focusing on incremental and cooperative improvements. We tested the framework flexibility and connectivity by developing web-based phenology models and climate matching tools. We have collaboratively developed stochastic spread models for both plant pathogens and arthropods called the Pest or Pathogen Spread (PoPS) Model that leverages SAFARIS to streamline modelling complex spread scenarios (10, 11). The compartmentalized framework design allows us to collaborate with others and increase our mapping capacity to support PPQ by incorporating new models. These models were typically developed for specific species and regions and required the input of detailed data drivers and model parameterization by skilled analysts. While collecting the necessary data drivers is already a challenge, some models also involve time-consuming tasks including collecting knowledge and conducting multiple trials to establish the needed parameters (42). In many cases, plant protection agencies like PPQ must quickly respond to and develop protection plans for introductions and outbreaks of little-known species. Therefore, our goal is to develop and incorporate generic models with appropriately processed input drivers that can cover a wide range of species and be easily parameterized. To accomplish our goal, we continue to develop a framework that integrates several model types that can address a variety of questions.

## Data availability statement

Publicly available datasets were analyzed in this study. This data can be found here: https://safaris.cipm.info/.

## Author contributions

YT designed and created the SAFARIS framework, assessed OAB, parameterized spongy moth Phenology Model, and wrote the manuscript. AT aided with developing the SAFARIS framework, assessed OAB, and wrote those portions of the manuscript as well as aided with edits. KM assessed OAB and wrote the OAB assessment portion of the manuscript. All authors contributed to the article and approved the submitted version.
