# Supplementary material for: SAFARIS: a spatial analytic framework for pest forecast systems
Source: Front Insect Sci. 2023 Jul 6;3:1198355. doi: 10.3389/finsc.2023.1198355 (PMC10926409; doi:10.3389/finsc.2023.1198355)
Supplement: Supplementary file 1 [file DataSheet_1.pdf]

Supplemental Tables

Supplemental Table 1. Summary of the weather and climate datasets that are currently available within SAFARIS.

Product	Developer/web link	Coverage	Temporal Resolution	Spatial Resolution	Variables[1]
**PRISM**	Oregon State University http://prism.oregonstate.edu/	Contiguous U.S.	Daily and Monthly since 1981 (near-real time)	4-kilometer	Temperature (avg, max, min) Precipitation Relative humidity
**DAYMET**	University of Montana Oak Ridge National Laboratory http://www.ntsg.umt.edu/project/daymet.php	Contiguous U.S. Mexico Southern Canada	Daily from 1980 to previous calendar year	1-kilometer	Temperature (max, min) Precipitation Relative humidity
**TopoWX**	University of Montana http://www.ntsg.umt.edu/project/topowx.php	Contiguous U.S.	Daily from 1948 to 2016	800-meter	Temperature (max, min)
**METDATA**	University of Idaho https://www.sciencebase.gov/catalog/item/54dd5df2e4b08de9379b38d8	Contiguous U.S.	Daily since 1979 (near-real time)	4-kilometer	Temperature (max, min) Precipitation
**NDFD**	National Oceanic and Atmospheric Administration https://www.ncdc.noaa.gov/data-access/model-data/model-datasets/national-digital-forecast-database-ndfd	Contiguous U.S. Hawaii Guam Puerto Rico Virgin Islands Alaska North Pacific Ocean Islands	7 days forecast	5-kilometer (cont. US) 2.5-km (HI, GU) 1.25-km (PR, VI) 6-km (AK) 10-km (NPOI)	Temperature Precipitation
**CFSR**	National Oceanic and Atmospheric Administration https://climatedataguide.ucar.edu/climate-data/climate-forecast-system-reanalysis-cfsr	Global	Hourly from 1979 to 2010	38-kilometer (Temp) 55-km (Precip)	Temperature Precipitation
**CFSv2**	National Oceanic and Atmospheric Administration http://cfs.ncep.noaa.gov/	Global	Hourly since 2011 (near-real time)	20-kilometer (Temp) 55-km (Precip)	Temperature Precipitation

[1] Some parameters are derived products. For example, we calculate relative humidity using various climate variables provided by each product.

Product	Developer/web link	Coverage	Temporal Resolution	Spatial Resolution	Variables[1]
**ERA5-Land**	European Centre for Medium-Range Weather Forecast https://www.ecmwf.int/en/era5-land	Global	Hourly from 1950 to previous calendar week	9-kilometer	Temperature Precipitation
**MACAv2-Livneh (20 GCMs)**	University of Idaho (downscaling method) https://climate.northwestknowledge.net/MACA/	Contiguous U.S.	Daily from 1950 to 2005 (historical base line) 2006-2099 (RCP 4.5 and 8.5)	Downscaled to 6-kilometer	Temperature (max, min) Precipitation

Supplemental Table 2. Summary of the models, analytic tools, and pest products that are currently available within SAFARIS

**Models: https://safaris.cipm.info/safarispestmodel/StartupServlet?safarismodels**			
**Models**	**Outputs**	**Weather Data**	**User Inputs**
Phenology Model	Degree day model of pest development.	Daily data at the continental United States, North America or global scales.	Start dates and end dates. Standard degree day model parameters, temperature thresholds and total degree days needed for the development of each pest life stage.
Pest or Pathogen Spread Model (PoPS) (SAFARIS provides a link to the PoPS model. It is run externally using data drivers within SAFARIS.)	Model of pest spread across a landscape in discrete time steps.	Weekly data at North America scale.	Historical pest detection data (i.e., latitude and longitude) and pest host distributions. Users can add additional dispersal information such as rail network and/or trade movements based on the nature of the pests.
MACA Phenology Model (Registered Users)	Climate change forecasts for pest phenology	20 MACA-downscaled General Circulation Models (GCMs). User can select up to five GCMs at a time. Two Relative Concentration Pathways (RCP 4.5 and RCP 8.5).	Start dates and end dates. Forecast range of years. Standard degree day model parameters, temperature thresholds and total degree days needed for the development of each pest life stage.
**Analytic Tools: https://safaris.cipm.info/safarispestmodel/StartupServlet?safaristools**			

**Analytic Tools**	**Outputs**	**Weather Data**	**User Inputs**
Plant Hardiness Zones	Climate matching for quick assessment of global suitability.	Two weather drivers and two 30-year time ranges.	None necessary. Optional: table of geographic coordinates of species records.
Temperature Mapping	Counting the number of days (consecutive or non-consecutive) that certain temperature requirements are met or a binary map showing whether areas met stated requirements.	Daily data at the continental United States, North America or global scales.	Start dates and end dates. Mean, maximum, or minimum temperature thresholds of interest.
Precipitation Mapping	Accumulation of precipitation over a specified time frame or a binary map showing whether areas met stated precipitation range requirements.	Daily data at the continental United States, North America or global scales.	Start dates and end dates, an optional range specification.
Relative Humidity Mapping	Counting the number of days (consecutive or non-consecutive) that certain relative humidity requirements are met or a binary map showing whether areas met stated requirements.	Daily data at the continental United States or North America scales.	Start dates and end dates, an optional range specification.
Temperature-Relative humidity Mapping	Counting the number of days (consecutive or non-consecutive) that certain temperature and relative humidity requirements are met or a binary map showing whether areas met stated requirements.	Daily data at the continental United States or North America scales.	Start dates and end dates. Mean, maximum, or minimum temperature and relative humidity thresholds.
Windrose	Graphical chart of the wind speed and direction at a specific location.	Daily data from weather stations across the globe.	Start dates and end dates, weather station of interest.

Climate Change Temperature Mapping (Internal)	Counting the number of days (consecutive or non-consecutive) that certain temperature requirements are met.	20 MACA-downscaled General Circulation Models (GCMs) of daily weather data. User can select up to five GCMs at a time. Two Relative Concentration Pathways (RCP 4.5 and RCP 8.5).	Forecast years and optionally, months. Start dates and end dates. Maximum or minimum temperature thresholds of interest.
Climate Change Precipitation Mapping (Internal)	Accumulation of precipitation over a specified time frame.	20 MACA-downscaled General Circulation Models (GCMs) of daily weather data. User can select up to five GCMs at a time. Two Relative Concentration Pathways (RCP 4.5 and RCP 8.5).	Forecast years and optionally, months.
**Pest Products: https://safaris.cipm.info/safarispestmodel/StartupServlet?pestinfo**			
**Pest Products**	**Description**		
Field Operations weekly phenology maps	The Field Operations weekly phenology maps indicate current pest stages using phenology models and near-real time weather data. The States and National phenology maps for 23 species are automatically generated on Wednesdays in the pdf format.		
PestCAST	PestCAST is a pest forecast system that indicates current pest stages and expected pest stages for the next 7 and 30 days using near-real time weather data, 7-day forecasts, and the 20-year historical weather data. There are currently 7 arthropod species in PestCAST.		
CAPS climate suitability maps	The CAPS climate suitability maps for pest surveillance maps support survey planning and prioritization for CAPS priority pest. Each map shows areas in which the climate is suitable for a pest to establish, given available information on the pest’s biology, development, and weather data over the last 20 years.		
